# Adenoid Cystic Carcinoma/Basal Cell Carcinoma of the Prostate: Overview and Update on Rare Prostate Cancer Subtypes

**DOI:** 10.3390/curroncol29030152

**Published:** 2022-03-09

**Authors:** Salvatore Cozzi, Lilia Bardoscia, Masoumeh Najafi, Andrea Botti, Gladys Blandino, Matteo Augugliaro, Moana Manicone, Federico Iori, Lucia Giaccherini, Angela Sardaro, Cinzia Iotti, Patrizia Ciammella

**Affiliations:** 1 Radiation Oncology Unit, Azienda USL-IRCCS di Reggio Emilia, 42123 Reggio Emilia, Italy or salvatore.cozzi@ausl.re.it (S.C.); gladys.blandino@ausl.re.it (G.B.); matteo.augugliaro@ausl.re.it (M.A.); moana.manicone@gmail.com (M.M.); federico.iori@ausl.re.it (F.I.); lucia.giaccherini@ausl.re.it (L.G.); cinzia.iotti@ausl.re.it (C.I.); patrizia.ciammella@ausl.re.it (P.C.); 2 Radiation Oncology Unit, S. Luca Hospital, Healthcare Company Tuscany Nord Ovest, 55100 Lucca, Italy; 3 Department of Radiation Oncology Shohadaye Haft-e-Tir Hospital, Iran University of Medical Science, Teheran 1449614535, Iran; masy.najafi@gmail.com; 4 Medical Physics Unit, Azienda USL-IRCCS di Reggio Emilia, 42123 Reggio Emilia, Italy; andrea.botti@ausl.re.it; 5 Interdisciplinary Department of Medicine, Section of Radiology and Radiation Oncology, University of Bari Aldo Moro, 70124 Bari, Italy; angela.sardaro@uniba.it

**Keywords:** adenoid cystic carcinoma, basaloid cell carcinoma, prostate cancer, surgery, radiotherapy, rare tumor variants

## Abstract

Adenoid cystic carcinoma/basaloid cell carcinoma of the prostate (ACC/BCC) is a very rare variant of prostate cancer with uncertain behavior. Few cases are reported in the literature. Data on treatment options are scarce. The aim of our work was to retrospectively review the published reports. Thirty-three case reports or case series were analyzed (106 patients in total). Pathological features, management, and follow-up information were evaluated. Despite the relatively low level of evidence given the unavoidable lack of prospective trials for such a rare prostate tumor, the following considerations were made: prostate ACC/BCC is an aggressive tumor often presenting with locally advanced disease and incidental diagnosis occurs during transurethral resection of the prostate for urinary obstructive symptoms. Prostate-specific antigen was not a reliable marker for diagnosis nor follow-up. Adequate staging with Computed Tomography (CT) scan and Magnetic Resonance Imaging (MRI) should be performed before treatment and during follow-up, while there is no evidence for the use of Positron Emission Tomography (PET). Radical surgery with negative margins and possibly adjuvant radiotherapy appear to be the treatments of choice. The response to androgen deprivation therapy was poor. Currently, there is no evidence of the use of truly effective systemic therapies.

## 1. Introduction

Adenoid cystic carcinoma (ACC) of the prostate, also called basaloid carcinoma and adenoid cystic-like tumor, was first described in 1974 as a rare but distinctive variant of prostatic adenocarcinoma. It is histologically identical to adenoid cystic carcinoma of the salivary glands [1]. ACC is typically a salivary gland tumor that is composed of ductal and myoepithelial cells, but it can also arise in different sites, including the skin, cervix, and breast [2,3,4,5].

For a long time, two reasons have been considered for the independent existence of this tumor: first, myoepithelial cells are not indigenous to the prostate, and second, adenoid cystic morphology occurs along a spectrum of basaloid proliferations that encompass basal cell hyperplasia, basal cell adenoma, and basal cell carcinoma. Finally, in 2016, *WHO Classification of Tumours of the Urinary System and Male Genital Organs* categorized adenoid cystic hyperplasia carcinoma and basaloid variants as malignant basal cell tumors (BCC) [6].

ACC/BCC is an extremely rare variant that is histologically difficult to detect, with uncertain behavior and about 100 cases reported in the literature compared to over 1 million acinar prostate cancer diagnoses every year. The age of onset ranges from 28 to 97 years, with peak incidence between 60 and 75 years; however, cases of young adults have been reported. When occurring in the prostate, these tumors predominantly show local infiltrative behavior.

Because of the rarity of this disease, therapeutic options for patients with ACC/BCC of the prostate are scarce. Most patients are treated with hormone therapy, radiotherapy, radical prostatectomy, or a combination of these treatments, although outcomes remain poor.

We retrospectively reviewed the published reports available in the literature until the present day. The used keywords for the literature research included “adenoid cystic”, “adenoid cystic-like”, “basaloid”, “basal cell carcinoma”, and “prostate”. Available clinical information, management, outcomes, and follow-up data were extracted. 

The management and follow-up data were also reviewed to ascertain the available treatment options for this rare type of prostate cancer.

## 2. Pathological Features

A wide range of basal cell lesions of the prostate gland have been described in the literature, from benign basal cell hyperplasia (BCH) to various infiltrative and invasive patterns. It is believed that, in contrast to usual prostate malignancies, they are originated from basal/reserve cells [7]. Grossly, ACC/BCC is commonly reported as a yellow specimen with a hard consistency. There are lobules separated by fibrous septa. There may be also hemorrhage, necrosis, and cystic changes [8]. Basophilic mucinous secretions are sometimes seen [7].

Histologically, prostate ACC has been identical to the usual head and neck ACCs, including the evidence of extensive perineural invasion [7,8,9,10]. Upon microscopic examination, the reported patterns are trabecular, glandular, cribriform, and variably sized solid nests. Early pathology reports already described ACC/BCC as irregular, variably sized nests of tumor cells, predominantly basaloid cells, and a lesser number of larger cells with pale eosinophilic cytoplasm, infiltrating the stroma with prominent cribriform architecture. Moreover, McKenny et al. observed an extensive intraglandular hyalinization completely replacing the glandular structures in some tumoral foci, different from the basaloid carcinoma that showed infiltration between normal glands, extraprostatic extension, and perineural invasion, but not with a cribriform pattern [11].

Tumoral cells have shown a high nuclear to cytoplasmic ratio, open chromatin, and scant cytoplasm with cytoplasmic vacuoles that resemble myoepithelial cells [7,8]. Basaloid characteristics in the reported cases are very prominent. Immunohistochemical examinations have shown a relationship among adenoid cystic carcinoma, basal cell hyperplasia, and adenoid basal cell tumors. Indeed, some authors have argued that it probably did not originate from the secretory epithelium of the prostate gland. Despite this, imaging, clinical, and pathologic evidence support ACC/BCC location to be within the prostate glandular tissue [9,10]. Beyond the perineural invasion (similar to head and neck ACCs), infiltrative pattern growth and extra-prostatic extension are also common features [7,8,10]. Since they originate from the basal cell layer, other than secretive, glandular epithelium, the almost or true negative Prostate-specific antigen (PSA) immunohistochemical staining is one of the usual features of ACC/BCC, except for some positive cases, especially in mixed-form ACC/BCC plus acinar adenocarcinoma [12,13].

From a molecular point of view, loss of PTEN expression and overexpression of EGFR are two frequent findings in ACC/BCC. The MYB translocation has often been described in true ACCs. In particular, the MYB–NFIB fusion protein has been reported to be associated with morphologic features reminiscent of adenoid cystic carcinoma in a cohort of basal cell carcinomas of the prostate [7]. Proliferative index, which is usually higher than 20% in ACC, may be helpful to distinguish BCH from ACC. Diffuse Bcl-2 staining in ACC may also help to differentiate ACC and BCH or usual PCs in which such evidence is absent or scant. CK7 protein is positive in the luminal part of ACC, while high-molecular-weight cytokeratins (i.e., HMCK, 34βE12) are positive in the peripheral parts of the tumor mass. P63 protein usually also results strongly positive in ACCs. S100 and PSA markers may be positive, but not in all cases [7,14]. Τhis tumor entity is probably not an homogenous tumor type comprised different subtypes and an in-depth molecular analysis could allow not only a better characterization of the disease but provide prognostic and predictive data of extreme importance. To date, from the data that emerged from our work, we are unable to state whether the presence of MYB translocation or HER2/PTEN alteration can allow different therapeutic approaches.

Compared to conventional acinar PCs, basal cell carcinoma usually shows little or no androgen receptor (AR) expression, with a very low percentage of patients or weak and patchy immunohistochemical staining reported in the literature [15]. The main pathological characteristics and hormonal reactions in ACC/BCC are summarized in Table 1.

## 3. Diagnosis and Staging

This rare form of prostate cancer tends to have non-specific symptoms that last for many years, showing an indolent course, for which it was originally suggested to be a potentially indolent disease. Most patients had symptoms of urinary tract obstruction, hematuria, nocturia, and pollakiuria or pelvic pain, often leading to incidental diagnosis by transurethral resection of the prostate (TURP). The initial step towards clinical diagnosis and staging was a digital rectal examination, which detected abnormalities in prostate glands. No preoperative imaging technique has provided sufficiently specific results. In some cases, anechoic lesions were observed by TRUS and typical of this cancer; however, these were not sufficient to establish a diagnosis between ACC/BCC and other prostate cancer subtypes or benign prostatic hyperplasia (BPH). Therefore, a pathologic examination of surgical or biopsy materials was required in order to obtain a certain diagnosis. It should be emphasized that, with the exception of a few cases (Table 2), the Prostate-specific antigen (PSA) level remained within the normal range, so it cannot be considered an index of tumor aggressiveness. These findings suggest that ACC/BCC of the prostate lacks the capability of PSA production and concomitant acinar ADK of the prostate was found in patients with PSA elevation [8]. The low PSA value could support the idea that this rare variant should not be considered and treated as prostate cancer. In our opinion, this consideration is incorrect since the PSA value in patients diagnosed with rare forms of non-acinar prostate cancer, such as ductal carcinoma of the prostate, was often in the normal range.

Normal PSA values, in addition to mild symptoms, pushed urologists to use medical treatments for benign pathology, probably delaying the cancer diagnosis. From the analysis of the literature, in almost all cases it is presented with locally advanced disease, with encroachment beyond the prostate capsule and infiltration of the bladder, rectum, and sometimes the pelvic wall or pelvis bone. Less than 10% of patients had stage IV disease at onset; however, 30–40% of patients developed a recurrence or metastases early after radical treatment, predominantly to the bone, liver, and lung [16,17]. 

However, these data could be underestimated considering the lack of sufficient follow-up time (median 1 year). Dong et al. reported a case of massive lung metastases after one year of radical prostatectomy (RP) who underwent multiple chemotherapy lines and had stable disease after treatment with etoposide [18].

Because of this potential aggression and the risk of developing early metastases, many authors suggested pre-treatment staging with Computed Tomography (CT scan), Magnetic Resonance Imaging (MRI), and possibly bone scan, as well as a follow-up CT scan every 3–6 months. In the early stages of the disease, according to Zang’s case report, MRI could be negative even when it is performed repeatedly [10].

There are no data available on the possible use of nuclear medicine tests for this tumor. Komura et al. described a case of stage IV ACC/BCC metastatic disease detected with 2-Fluoro-2-deoxy-D-glucose Positron Emission Tomography/Computed Tomography (PET/CT) [19]. Moreover, sporadic cases of positive metastases by ACC extra-prostatic tumors with 68 Ga-Prostate-Specific Membrane Antigen (PSMA) are reported. This could be a possible area for future investigation [20,21].

## 4. Treatment Options and Outcomes

Given the rarity of ACC/BCC prostate cancer, there were no prospective trials to determine the optimal treatment. Thirty-three articles published in the literature, for a total of 106 patients, were evaluated with the aim of identifying the most suitable treatment and evaluating outcomes (Table 2). Various treatment approaches have been described in the literature. They are mainly based on tumor histology and/or borrowed from some more usual ACC/BCC tumor sites (e.g., head and neck), hence highlighting there is no uniformity in the management of such a rare prostate disease due to the lack of knowledge on its clinical and biological characteristics. 

Follow-up data were reported in 28 of 30 articles (90 patients) and ranged from 3 to 136 months. Of these, four (4.4%) died from cancer-related causes, 41 (45.5%) were reported to be free of recurrence (NED), 21 (23.3%) were alive, but with evidence of disease, and 24 (26.6%) showed local progression or developed distant metastases mainly in the lungs, liver, and rarely bone. 

In some patients, radical surgical resection was a treatment option for localized disease, ensuring free margins and a clear histological characterization of ACC/BCC. However, the extensive locally infiltrative pattern and perineural spread often cause difficulty in achieving high tumor control rates [24,38,44]. Prognostic factors such as advanced tumors, positive resection margins, and perineural infiltration drove the indication for postoperative radiotherapy [37]. Such findings were imported from the guidelines for acinar prostate cancer and ACC cancer of the head and neck region. In fact, most of the patients (38 patients, 36.5%) were treated with TURP, acting immediately on the clinical symptoms, while in five (4.8%) cases, only diagnostic biopsy was performed. 

Aggressive surgical treatments, such as pelvic exenteration, that were initially applied (four patients, 3.8%) [16,22,39] were subsequently abandoned to give way to more conservative treatments, often characterized by the combination of two or more therapeutic approaches. Radical prostatectomy was adopted in 17 patients (16.4%), while postoperative radiotherapy, with a total dose of 66 Gy in 33 sessions or 45 Gy in 15 sessions, was added to RP in 11 cases (10.36%). Radical radiotherapy for a total dose of 45–50 Gy associated with hormone therapy or chemotherapy was administered in eight cases (7.9%).

The authors of this article believe it is important in the case of patients with urinary symptoms not to act immediately with TURP, but to opt for symptom control through the use of drugs since TURP could preclude the possibility of surgery or disallow escalation of the radiotherapy dose, compromising the patient’s outcome.

Iczkowski et al. [16] reviewed and reported 19 cases of ACC/BCC neoplasm of the prostate, identifying young age, involvement of peripheral zone of the prostate, extra-prostatic spread, and perineural and peri-glandular invasion as important prognostic factors. Ali and colleagues also suggested potentially aggressive behavior attributable to the common presence of cancer cells in the periprostatic adipose tissue and extension to the thick muscle bundles of the bladder neck [17]. 

As far as systemic therapy is concerned, it is not possible to draw clear conclusions. Only six (5.7%) patients with advanced ACC/BCC disease received systemic approaches, with various drugs used, such as 5-Fluorouracil plus Mitomycin C; Docetaxel; Carboplatin plus Paclitaxel; Cisplatin; and ADT (Bicalutamide and/or LHRH analogues/antagonists), alone or combination strategies [9,13,29,36,37]. A case of locally advanced prostate BCC treated with concurrent chemoradiation was recently reported. A 70 Gy total target dose was delivered in 35 fractions with an intensity-modulated technique and concomitant, weekly cisplatin with evidence of complete local remission and disappearance of the lower urinary tract symptoms and pain at the one-year follow-up visit, but distant metastatic oligoprogression of the disease [42]. Of note, since ACC/BCC has proven to not express the AR, the impact of ADT remains unclear, even if luteinizing hormone-releasing hormone agonists and antiandrogen drugs are still considered the treatment of choice for inoperable cases or metastatic disease [15,39]. The question arises spontaneously whether the forms of ACC/BCC of the prostate can or should be treated as similar tumors of other sites (i.e., ACC tumors of the head and neck area). In the some reports, the authors report experiences in adding chemotherapy based on cisplatin or taxanes (as in head and neck pathologies), but there are very few cases and it is not possible to draw a conclusion [13,33,42].

## 5. Discussion

Adenoid cystic carcinoma/basal cell carcinoma is an extremely rare variant of prostate cancer first described in 1974. Just over 100 cases are currently reported in the literature worldwide. In view of the rarity and the lack of prospective studies, it is possible to define some features only based on few case reports. Despite this, it is possible to draw some important conclusions which may guide physicians to choose the best therapeutic approach. The correct identification of ACC/BCC from a pathological point of view is of extreme importance and therefore, when there is histological suspicion, the specimens should be referred to an expert pathologist. The serum PSA value does not represent a useful marker in this histological variant, so a clinical picture with persistent obstructive urinary symptoms unresponsive to medical therapy, even in the absence of a rise in PSA, necessitates investigation with a biopsy. Above all, serum PSA should not be used as the sole tool for follow up. Contrary to what has been hypothesized in the past, ACC/BCC has usually been shown not to present with an indolent character, rather it tends to have loco-regional spread with potential to metastasize. Given the potential aggressiveness of the tumor, owing to metastatic disease at presentation, an adequate radiological staging with CT scan plus bone scan and MRI should be performed prior to radical treatment, with curative intent and during the follow-up period. If necessary, the use of second-level functional imaging (i.e., Choline-PET or PSMA-PET) is desirable to complete the staging framework [13,43]. Moreover, TURP should also be avoided as an upfront treatment in cases of symptoms in order not to compromise subsequent treatment and preferred medical treatment.

Young age, involvement of the peripheral zone of the prostate, extra-prostatic spread, and perineural and peri-glandular invasion are important prognostic factors. The presence of these factors make radical prostatectomy alone insufficient to guarantee disease control, therefore a combined treatment including adjuvant radiotherapy should be considered as a standard of care. No conclusive data can be drawn on the best systemic treatment, nor on the efficacy of androgen deprivation therapy, especially as ACC/BCC has proven to be independent from androgen stimulation [19,33]. Recently, a phenotypic multi-dimensional assay testing the patient’s tumor tissue against different drug combinations has been proposed, along with an accurate prediction of clinical outcome, thus offering a personalized way to select the most appropriate treatment option for the individual patient, especially with rare cancers [13,38]. A more accurate refinement of the histopathologic features of prostatic ACC/BCC can probably help tailor treatment based on tumor phenotypes and involved genetic pathways that includes various potential therapeutic targets [39]. Since prospective studies are not conceivable due to the rarity of ACC/BCC worldwide, the collection of real-world data, possibly with large international, retrospective databases is desirable to better understand the adequate management of ACC/BCC and the role of different therapeutic strategies.

## Figures and Tables

**Table 1:** Summary of the main pathological features of ACC/BCC.

Morphologic Characters	Immunostaining	Molecular Characteristic
Scare cytoplasm		loss of PTEN expression overexpression of EGFR MYB–NFIB fusion
High N/C ratio		
Irregular and angulated nuclei with open chromatin		
May exhibit nuclear and cytoplasmic micro vacuolation		
Infiltration of adjacent parenchyma		
BCC pattern:	BCC pattern:	
Variably sized, solid nests, cords or trabeculae, peripheral palisading of basaloid cell	Basal cell markers, p63 or HMCK (34βE12)	
ACC pattern:	ACC pattern:	
Prominent cribriform architecture	CK 20-/CD7+ staining	
Eosinophilic, hyaline, basement membrane-like material	CK7 in pure solid form	
	Basal cell nests	
	Bcl-2 strongly and diffusely +	
	High Ki67 nuclear staining	

Abbreviations: ACC: adenoid cystic carcinoma; BCC: basal cell carcinoma; N/C: nuclear-cytoplasmic ratio; HMCK: high-molecular-weight cytokeratins; CK cytokeratine; CD: cluster of differentiation; Bcl-2: B cell lymphoma-2; Ki67: marker of proliferation Ki67; ADT: androgen deprivation therapy.

**Table 2:** Summary of case reports and case series published in the literature.

Author (Year)	*N* of Pts * (Tot: 106)	Age	PSA (ng/mL)	Symptoms	Diasease Stage	Treatment	Outcomes
Frankel, K. 1974 [22]	1	69	/	Acute urinary retention and nocturia	cT1c	TURP	36 m fup: NED
Tannenbaum, M. 1975 [23]	2	/	/	/	cT4	/	/
Kramer, S.A. 1978 [24]	1	55		Perineal pain and tenderness	cT4	TURP plus Pelvic exenteratio + RT (60 Gy)	/
Kuhajda, F.P. 1983 [25]	1	66		Urinary obstruction	/	TURP plus RT	NED
Gilrnour, A.M. 1986 [26]	1	76	/	5 y history of nocturia and poor stream	Organ confined disease	TURP plus RP	8 m fup: NED
Ahn, K.S., 1991 [8]	1	38	/	Long history of nocturia and Dysuria	cT3b	RP	/
Denholm, S.W. 1992 [9]	1	28	Normal range	Urinary obstruction	cT4	TURP plus RT (45 Gy in 20 Fx) plus chemotherapy (5 Fluorouracil-Mitomycin C)	18 m fup: reduction of pelvic mass
Hasan, N. 1996 [22]	1	66	Normal range	Acute retention	Organ confined disease	TURP	4 m: NED
Pariente, J.L. 1998 [27]	1	73	168	/	/	TURP plus Androgen blockade	12 m fup: NED
Young, R.H. 1998 [28]	2	Case 1: 60 Case 2: 68	/	Acute retention	/	TURP and RP	8 y fup: NED
Minei, S. 2001 [29]	1	43	2	Urinary Obstruction	/	TURP	/
Schmid, H.P. 2002 [30]	1	43	Normal range	/	/	PR plus RT	8 y fup: local progression
Iczkowski, K.A. 2003 [16]	19	43–87	<9 ng/mL	Urinary Obstruction	4 cases: stage IV	TURP (10 pts), RP (2 pts), exenteratio, (2 pts) combined RP and RT (4 pts), biopsy (1 pts)	Mean fup 26 m (range 3–132): 10 pts: NED, 4 developed metastases 3 pts alive with tumor, 1 pt died of tumor
Mastropasqua, M.G. 2003 [14]	1	65	8.5	Nocturia, pelvic pain	pT3bN1	RP + LAD	8 mo fup: lung metastases
McKenney, J.K. 2004 [11]	4	36–60	/	/	Organ confined disease (1 pt) cT4 (3 pts)	RP (2 pts), TURP	1 died 3 mo after PR 9 m fup: NED
Fayyad, L.M. 2006 [31]	1	75	/	/	/	TURP + CT + ADT	5 y fup: died for metastases
Ali, T. 2007 [17]	29	42–89	/	Urinary Obstruction	/	TURP (16 pts), TURP + RP (5 pts) RP + RT + CT (4 pts) Biopsia (4 pts)	Mean fup 4.3 y: 14 pts NED 4 pts local recurrence 4 pts metastases
Komura, K. 2010 [19]	1	67	Normal range	Urinary Obstruction, pelvic pain	IV	Docetaxel and Extramustine	Lung metastases after 3 m of treayment
Bohn, O.L. 2010 [32]	1	65	Normal range	Long history of dysuria and urinary outlet obstruction	Organ confined disease	RP	12 mo fup: NED
Ahuja, A. 2011 [33]	1	32	Normal range	Obstructive lower urinary tract symptoms	cT4	Bilateral orchidectomy and Bicalutamide	6 mo fup: Stable disease
Tuan, J. 2012 [34]	1	78	Normal range	Urinary tract symptoms, nocturia and gross hematuria	T4N1M0	TURP plus RT (45 Gy in 20 Fx) plus CT (5-Fluorouracil + Mitomycin C)	36 mo fup: NED
Stearns, G. 2012 [35]	1	69	Normal range	Hematuria	cT4N0	Etoposide and Cisplatin plus RP	Early progression
Chang, K. 2012 [36]	3	48–65	Normal range	Acute urinary retention	Cases 1 and 2: Organ confined disease Case 3: lung metastases	Cases 1 and 2: 50 Gy RT Case 3: Androgen blockade (Bicalutamide + Goserelin)	Cases 1 and 2: bone progression after 2 mo Case 3: died 5 mo after treatment
Tsuruta, K. 2012 [37]	1	48		Hematuria	cT4	Etoposide and Cisplatin plus pelvic exenteratio	Liver metastases after 3 mo
Bishop, J.A. 2015 [38]	12	65–86	/	/	/	TURP	/
Simper, N.B. 2015 [39]	9	57–97	/	/	Locoregional confined disease,	TURP (6 pts), Pelvic exenteratio (1 pt), RP (2 pts)	44 mo fup: 5 pts NED 4 pts local recurrence 1 pt metastases
Zang, M. 2016 [10]	1	73	1.9	Nine years of peritoneal pain	cT4	Pelvic exenteratio	22 mo fup: PSA:0
Bernhardt, D. 2018 [40]	2	Case 1: 65 Case 2: 44	Normal range	Perirectal pain	Case 1: pT2c pN0 M1, Case 2: cT4	TURP plus RP plus RT as photon IMRT plus C12 heavy ion boost	Case 1: 16 mo fup local and distant progression Case 2: NED
Shibuya, T. 2018 [41]	1	68	Normal range	/	cT1c	RP	1 y fup: NED
Dong, S. 2020 [18]	1	62	Normal range	/	pT2	RP + RT	2 y fup: lung metastases
Julka, P.K. 2020 [13]	1	79	/	Hematuria	CT4N0M1(liver)	TURP plus CT (Carboplatin + Paclitaxel) then ADT (Degarelix)	16 mo fup: stable disease
Ridai, S. 2021 [42]	1	40	3.5	Obstructive lower urinary tract symptoms	cT3b	TURP plus concurrent CT (Cisplatin)-RT as photon IMRT	1 y fup: cerebellar metastases
He, L. 2021 [43]	1	92	<0.05 post-TURP	Urethral stricture, urinary retention	cT1c	TURP plus RT	4 mo fup: NED

* Abbreviations: N°: number; PTS: patients; PSA: Prostate-specific antigen; TURP: transurethral resection of prostate; RP: radical prostatectomy; fup: follow-up; MO: months; Y: years; RT: radiotherapy; C12: 12 carbon; IMRT: Intensity-modulated radiation therapy; NED: no evidence of disease, ADT: androgen deprivation therapy; CT: chemotherapy. The text continues here (Table 2).

## Data Availability

Not applicable.
